# Neurospecific fabrication and toxicity assessment of a PNIPAM nanogel encapsulated with *trans*-tephrostachin for blood-brain-barrier permeability in zebrafish model

**DOI:** 10.1016/j.heliyon.2022.e10237

**Published:** 2022-08-18

**Authors:** Pitchai Arjun, Jennifer L. Freeman, Rajaretinam Rajesh Kannan

**Affiliations:** a Neuroscience Lab, Centre for Molecular and Nanomedical Sciences (CMNS), Centre for Nanoscience and Nanotechnology (CNSNT), School of Bio and Chemical Engineering, Sathyabama Institute of Science and Technology, (Deemed to Be University) Jeppiaar Nagar, Rajiv Gandhi Salai, Chennai, 600119, Tamil Nadu, India; b School of Health Sciences, Purdue University, West Lafayette, IN 47907, USA

**Keywords:** PNIPAM Nanogel, Polysorbate 80, *trans*-Tephrostachin, Neurospecific, Zebrafish, Toxicology, Alzheimer's disease, Behavior

## Abstract

Biocompatible Poly(N-isopropylacrylamide) (PNIPAM) nanogels (NGs) were developed at 40–65 nm to deliver *Trans*-Tephrostachin (TT) in zebrafish brain. Neurospecific PNIPAM NGs are functionalized with polysorbate 80 (PS80) to overcome the Blood Brain Barrier (BBB). The TT loaded with NG (NG + TT) was confirmed in UV-spectroscopy and transmission electron microscopy (TEM) with 90% efficiency of controlled release at 37 °C. The neurospecificity of NG was confirmed in 144 hours post fertilization (hpf) larvae with PS80 surface-treated rhodamine-B (Rh–B) conjugated NG and visualized in the zebrafish CNS. Oral gavaging of TT loaded NG with PS80 surface treatment (NG + TT + PS80) was confirmed to cross the BBB in adult zebrafish at 37 °C. TT release was detected by RP-HPLC. LC_50_ was determined as 250 μg/ml for NG, 172 μg/ml for NG + TT, and 0.9 μg/ml for TT at 96 hpf and confirmed the lesser toxicity in TT bound NG. Delays in growth and malformations were observed at concentrations above the 96 hpf-LC_50_. The behavior outcomes were varied with phase - and concentration-dependent hypo- or hyperactivity. The altered expression of genes associated with Alzheimer’s disease (AD) was found at 96 hpf of its LC_50_ concentration. The expression of *appa* was significantly increased for TT and supporting the TT to bind NG without altering the AD genes. Thus the study suggests the biocompatible potential of PNIPAM and its neurospecific delivery to the brain.

## 1 Introduction

The development of any drugs targeting the Central Nervous System (CNS) takes longer to develop. Clinical trials of CNS drugs are challenging due to the impermeability of the blood-brain barrier (BBB) [1]. It acts as a checkpoint for the entry of selected molecules from the blood [2]. Capillary endothelial cells are interconnected by tight intercellular junctions in BBB to prevent the entry of molecules [3]. 2% of lipophilic small molecules are capable to cross BBB [4]. Specifically designed nanoparticles have the potential to cross the BBB for absorption [5]. Receptor-mediated transcytosis is a non-invasive drug delivery in CNS with lower side effects [6]. One of the most important developments in pharmaceutical research is the creation of a drug delivery system to cross the BBB by conjugating the nanoparticles with surface ligand [7].

Polymer nanoparticles with 1–1000 nm have been grown rapidly for clinical medicine [8] and extended in the circulation system as drug delivery vehicles [9]. NGs can react with external stimulants, biological agents, and chemicals [10]. Due to their excellent thermal sensitivity the poly(N-isopropylacrylamide) (PNIPAM) has lower critical solution temperature (LCST) at 32 °C [11] for drug delivery applications [12]. PS80-containing Poly (n-butylcyanoacrylate) nanoparticles can reach the brain parenchyma B and E apolipoprotein receptors in the endothelial cells when administered systemically [13]. To tackle the BBB permeability, we conjugated PS80 with NG to promote the transmission of PNIPAM NG to cross the BBB.

Zebrafish (*Danio rerio*) is a complex vertebrate organism with higher homology to humans. Major neurotransmitters, hormones, receptors, and apolipoproteins of zebrafish are similar to humans [14]. Studies have shown that zebrafish BBB maturation occurs around 72 hpf with common functional physiology [16]. *Ex vivo* embryos can be used by real-time monitoring fluorescent probes and drug delivery systems (DDSs) [15]. Thus, the present study is aimed to develop a biocompatible NG for neurospecific drug delivery of TT for crossing the BBB and to characterize toxicity, behavioral responses and expression alterations of AD risk genes.

## 2 Methodology

### 2.1 Materials

N-isopropylacrylamide (NIPAM), N,N′-Methylenebisacrylamide (MBA), sodium dodecyl sulphate (SDS), potassium per sulphate (KPS), and Polysorbate 80 (PS80) were purchased from Sigma Aldrich Chemicals.

### 2.2 Preparation of nanogels

**Scheme 1:**
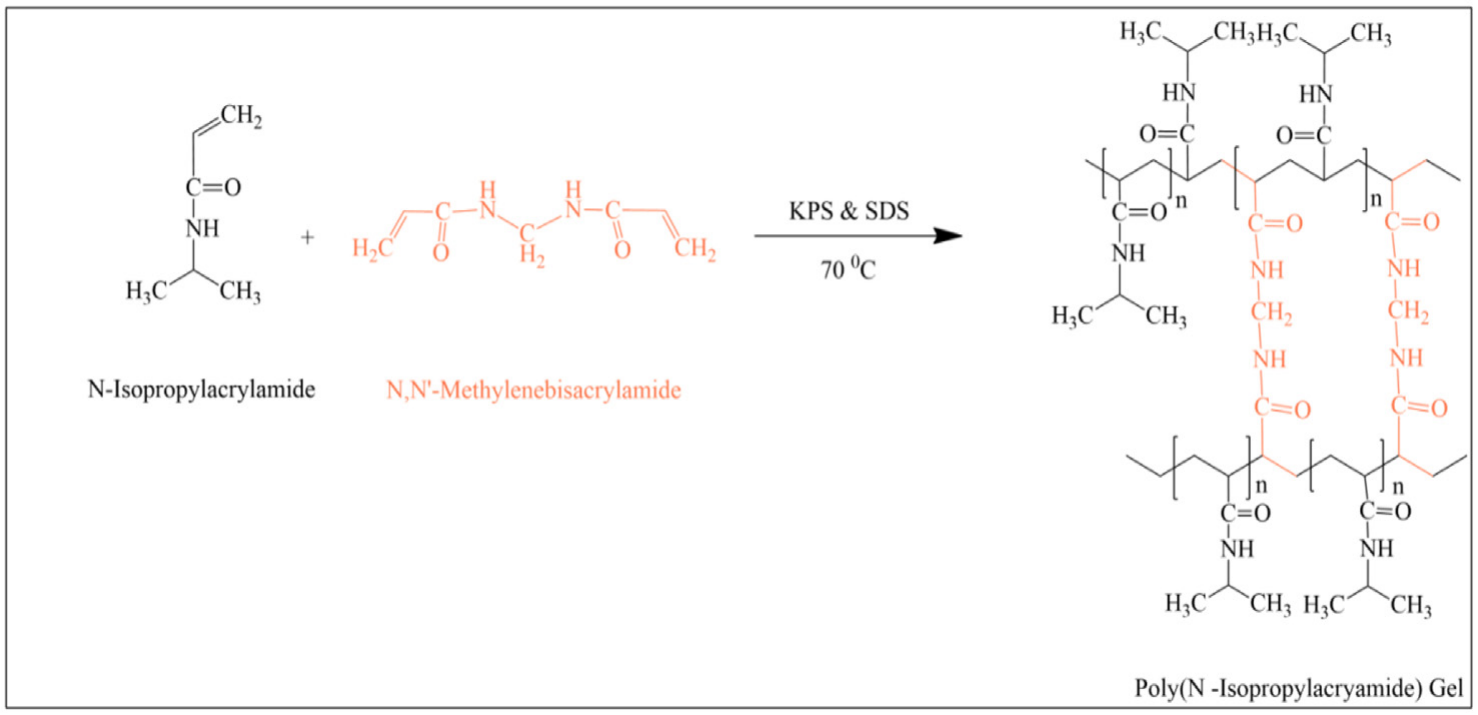
Scheme of PNIPAM-NGs synthesis.

P(NIPAM-MBA) NGs were synthesized by polymerization (Scheme 1). 50 ml H_2_O was pumped with N_2_ gas in a round bottom flask for 5 min to eliminate the dissolved oxygen. 1.13 g NIPAM (10 mmol), 0.0616 g MBA (0.4 mmol), 0.027 g KPS and. 0.27 g SDS were added and mixed in a magnetic stirrer. Polymerization process was initiated at 60 °C with 20-minute stirring at 500 rpm and chilled in cold-water. Further dialysis (8,000–14,000 molecular weight) was continued for a week and frozen for 48 h.

### 2.3 *Trans*-tephrostachin, Rh–B loading, and conjugation of PS80 with NG

10 mg of lyophilized NG was dispersed into 10 ml of double distilled water. Then TT or Rh–B solution in DMF (10 mg ml^−1^) was slowly added into the co-polymeric solution and stirred at room temperature for TT or Rh–B entrapment process and lyophilized for further use. PS80 was added along with TT or Rh–B loaded NG for 1% coating. The nanogels and conjugates were characterized by Field Emission Scanning Electron Microscopy (FESEM) (Supra-55, Carl Zeiss, Germany) Transmission Electron Microscopy (TEM) (JEOL, 2100F). Fourier Transform Infrared (FTIR) (FTS-135, Bio-Rad, USA). Dynamic Light Scattering (DLS) (Malvern Instruments, Worcestershire, UK).

### 2.4 Entrapment efficiency (E %)

The entrapment capacity (E %) of TT loaded in NG was determined. The NGs were removed from the unentrapped (free) TT using a NANOSEP (100 kD cut off) membrane filter and the amount of free TT was measured as

E% = (TT_total_ - TT_free_)/TT_total_∗100

Where, TT_total_ was the total amount of TT loaded in the NGs and TTfree was the total amount of unentrapped TT in the solution.

### 2.5 *In vitro* release studies

The cumulative release of TT from NG in 0.1 M Phosphate buffered saline (PBS) (pH 7.4) was measured by dialysis bag technique. The solution was centrifuged at 3000 rpm for 10 min to separate the released TT from the NGs and dissolved in 1 ml of CHCl_3_. The absorbance was measured at 269 nm and substituted with an equivalent amount of fresh PBS to hold the sink. Then, the released drug concentration was measured using the regular TT curve in CHCl_3_. The percentage of TT released was determined as:

Release (%) = [TT] release/[TT] total/100

Where [TT] _release_ was the concentration of released TT collected at time t and [TT] _total_ was the total amount of TT entrapped in the nanoparticles.

### 2.6 Determination of cytotoxicity

The mouse primary fibroblast cells were purchased from National Center for Cell Science, Pune, India. The cells were grown in 96-well cell culture plates until 80% confluent. The culture media was supplemented by a fresh medium containing diluted NGs in 0.1% DMSO in full medium and the cells were exposed to the NG, NG + TT, or TT for 24 h. The MTT assay was carried out. MTT (3-(4,5-dimethyl-2-thiazoyl)-2,5- diphenyltetrazolium bromide, Sigma, 250 μg/mL final concentration) was added to the cells for 2 h and measured at 540nm (Ensight Multimode reader PerkinElmer).

### 2.7 Evaluation of cell uptake of nanogels

Fibroblast cells were grown until 80% confluency in 24-well culture plates. The culture medium was replaced with a medium containing NG + Rh–B diluted at 14% (v/v, final concentration) and the cells were exposed to the NGs. Further the cells were washed with PBS and Rh–B fluorescence was observed.

### 2.8 Zebrafish husbandry

Adult zebrafish 5D strain was maintained in a recirculating stand-alone system (Aquaneering, USA or Aquatic Habitats, USA) at 28 °C on a 10:14 h dark: light cycle with a pH range of 7.1–7.3 and conductivity of 470–550 μS. Fishes were bred to attain embryos for experiments in breeding tanks [17]. All protocols were reviewed and approved by the Institutional Ethical Committee (approval number for animal usage IBSC/2013/DBT-IDB/RRK-009) of Sathyabama University and Purdue University (protocol #1110000088).

### 2.9 Neurospecific analysis by microinjection in zebrafish larvae

Nanogels were injected in the aortic region by microinjections to analyze the neurospecificity of the NG at 144 hpf zebrafish. The coating with PS80 permitted the crossing of NG through the BBB. The toxicity of PS80 was previously established in zebrafish and optimized in this study [18]. 5 nl of NG, NG + Rh–B, or NG + Rh–B + PS80 were injected with a Femtojet microinjector (Eppendorf). The retrospectivity was analyzed with a Leica MZ16FA stereo fluorescent microscope.

### 2.10 RP-HPLC analysis of drug release in adult zebrafish

Five Adult zebrafish were taken in each batch for the administration of TT, NG + TT, NG + TT + PS80, or negative control (water only). Venflon syringe of 24G was used to administer 5 μl volume of the drug to adult zebrafish by inserting the plastic needle 1 cm below the gills to reach the end of the esophagus [19]. These fishes were kept 1min in warm water above LCST 37 °C for the release of TT from NGs until regaining their original physiological function from the anesthetic condition and the concentrations were determined after effective dosage calculations. The oral treatment was continued for a week as one time dosage. The orally treated fish brain was dissected and homogenized in 500 ml of methanol. This sample was centrifuged at 14,000 rpm for 15 min at room temperature and the supernatant was analyzed with HPLC as previously described [18].

### 2.11 Fish embryo toxicity

Fish Embryo Toxicity (FET) was carried out as per the OECD guidelines (OECD, 2012). Embryos were distributed in a 24 well plate containing 3 mL of the culture water with several dilutions of NG (200, 220, 240, 260, 280, or 300 μg/ml), NG + TT (150, 175, 200, 220, 240, or 250 μg/ml), and TT (0.2, 0.5, 0.8, 1.0, 1.2, 1.4, or 1.6 μg/ml) respectively and LC50 were calculated at 96 hpf. The quantitative data were expressed as mean ± SD using GraphPad Prism version 9.11 (GraphPad Software Inc., San Diego, USA).

### 2.12 Behavior analysis

Locomotor behavior was assessed at 120 hpf in larvae with the visual motor response using Noldus *Danio* vision Observation Chamber following exposure to NG (214, 250, or 285 μg/ml), NG + TT (143, 172, or 200 μg/ml), or TT (0.8, 0.9, or 1.0 μg/ml) for 4 to 120 hpf. Each treatment group within a biological replica consisted of twenty-four larvae (subsamples). Following a 10 min dark acclimation period, the Noldus white light routine was used to test visual motor response by exposing larvae to 10 min intervals by alternating light and dark periods for 50 min in a previous study [20]. Statistical analysis was evaluated using ANOVA on SAS Statistical Software (SAS Institute Inc., Cary, NC) to determine differences among groups by phase (α = 0.05).

### 2.13 Transcript analysis of AD associated genes by qPCR

A set of genes associated with AD (*apoea*, *apoeb*, *appa*, *appb*, and *psen1*) were evaluated for expressions in larvae at 120 hpf with following exposures (4–120 hpf). 20 embryos per well in a 6 well plate were exposed to the 96 hpf-LC50s of the NG (250 μg/ml), NG + TT (172 μg/ml), TT (0.9 μg/ml), or aquaria water only as the negative control. A total of 3 biological replicates (n = 3) were collected. Each biological replicate consisted of a pool of 45–50 larvae. RNA was isolated and cDNA was synthesized following established protocols [21]. The relative expression of apolipoprotein Ea (*apoea),* apolipoprotein Eb *(apoeb),* amyloid precursor protein a *(appa),* amyloid precursor protein b *(appb),* and presenilin 1 (*psen1*) was determined via quantitative PCR (qPCR) method [22] and the primers of the corresponding genes are showed in Table 1 *gapdh* was chosen as a reference gene. Data were checked for normality and statistical significance by ANOVA and Fisher's least significant difference (LSD) atα = 0.05 using SAS Statistical Software (SAS Institute Inc., Cary, NC).

**Table 1:** Primers for qPCR analysis for NG, NG + TT, and TT.

Gene symbol	Primer sequence	
*apoea*		
	Forward:	gacacactgatctctgacagca
	Reverse:	Atcttcgttgaacttctgggct
*apoeb*		
	Forward:	ctaaggaacgcagcactcagta
	Reverse:	Cttcagtttgcgtgtgtaggtg
*appa*		
	Forward:	accgtctgctctcacactacta
	Reverse:	Tcagtgtgaggaggaagaggaa
*appb*		
	Forward:	ggcagatgtgtagaaggaagct
	Reverse:	Ccccatgcaaccattaagtgtg
*psen1*		
	Forward:	gcgttggtgtatagcgagtttc
	Reverse:	gcgagcattaacagtagcttgg
*Gapdh*		
	Forward:	Tctgacagtccgtcttgagaaa
	Reverse:	acaaagtgatcgttgagagcaa

## 3 Results

### 3.1 Preparation of the PNIPAM nanogel

**Figure 1:**
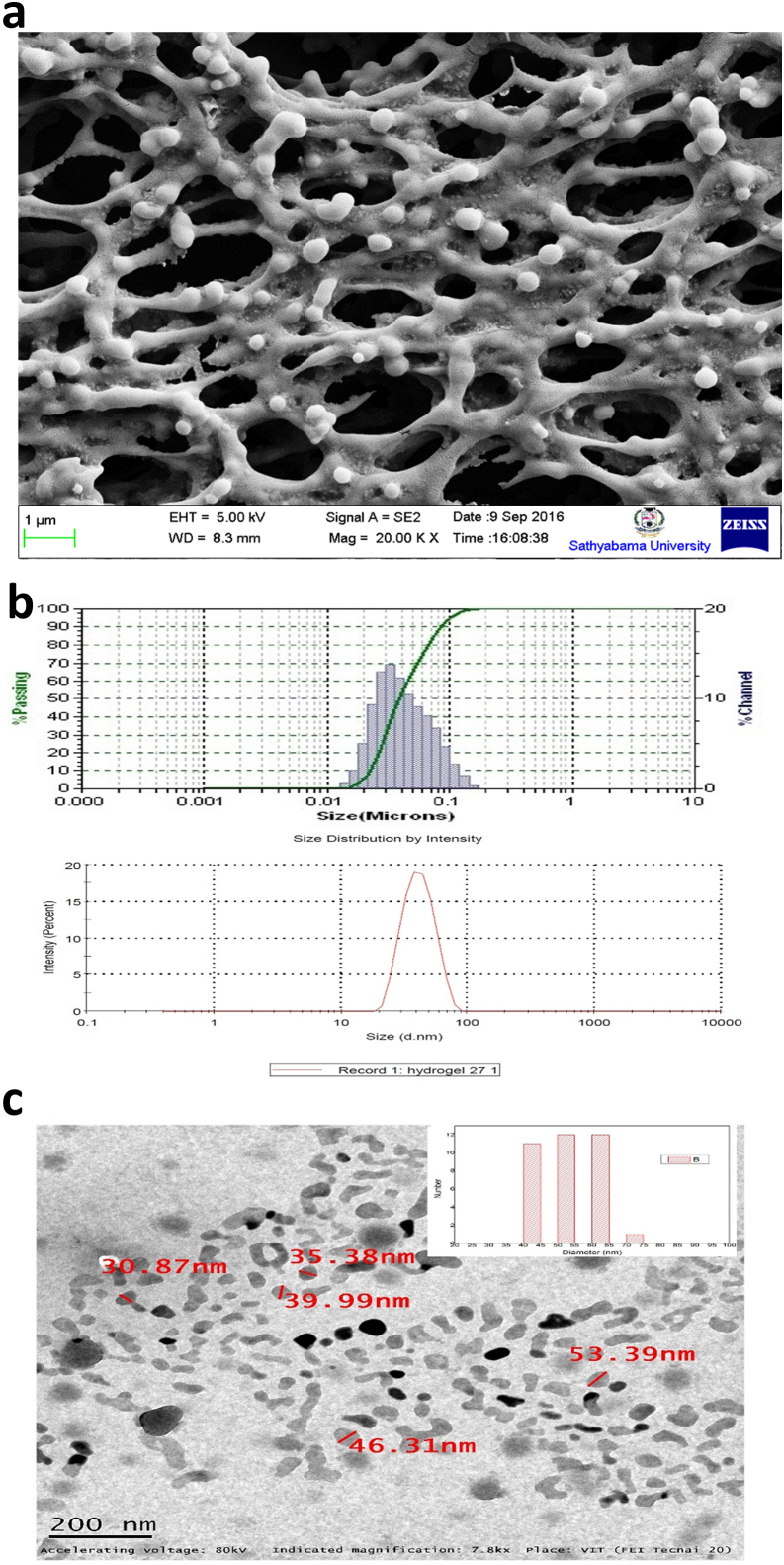
**a.** SEM morphology of the fabricated PNIPAM-NG shows the cross-linking and mesh like nature at 20 min polymerization time for 60 °C. Size of the synthesized NG. Both DLS (b) and TEM (c) images showed the particle size range from 40 to 65 nm.

FE-SEM micrographs demonstrated freeze-dried NG crosslink structures and distributed homogenously across the network (Figure 1a). The particle size was 40–65 nm, based on the DLS (Figure 1b) and TEM (Figure 1c). To correlate the optical response with conformational changes with the PNIPAM NG system, TEM was performed (Supplementary Figure 1) with the PNIPAM system. The increase in temperature above the LCST caused an increase in hydrodynamic diameter from 50 to 80 nm despite the polymer collapse inducing aggregation. The findings of the FT-IR identification showed that SDS was no longer found in the NGs after full water wash and unsaturated double bonds were no longer found in NGs (Supplementary Figure 2).

### 3.2 Loading of TT, Rhodamine-B, and conjugation of PS80 with NG

**Figure 2:**
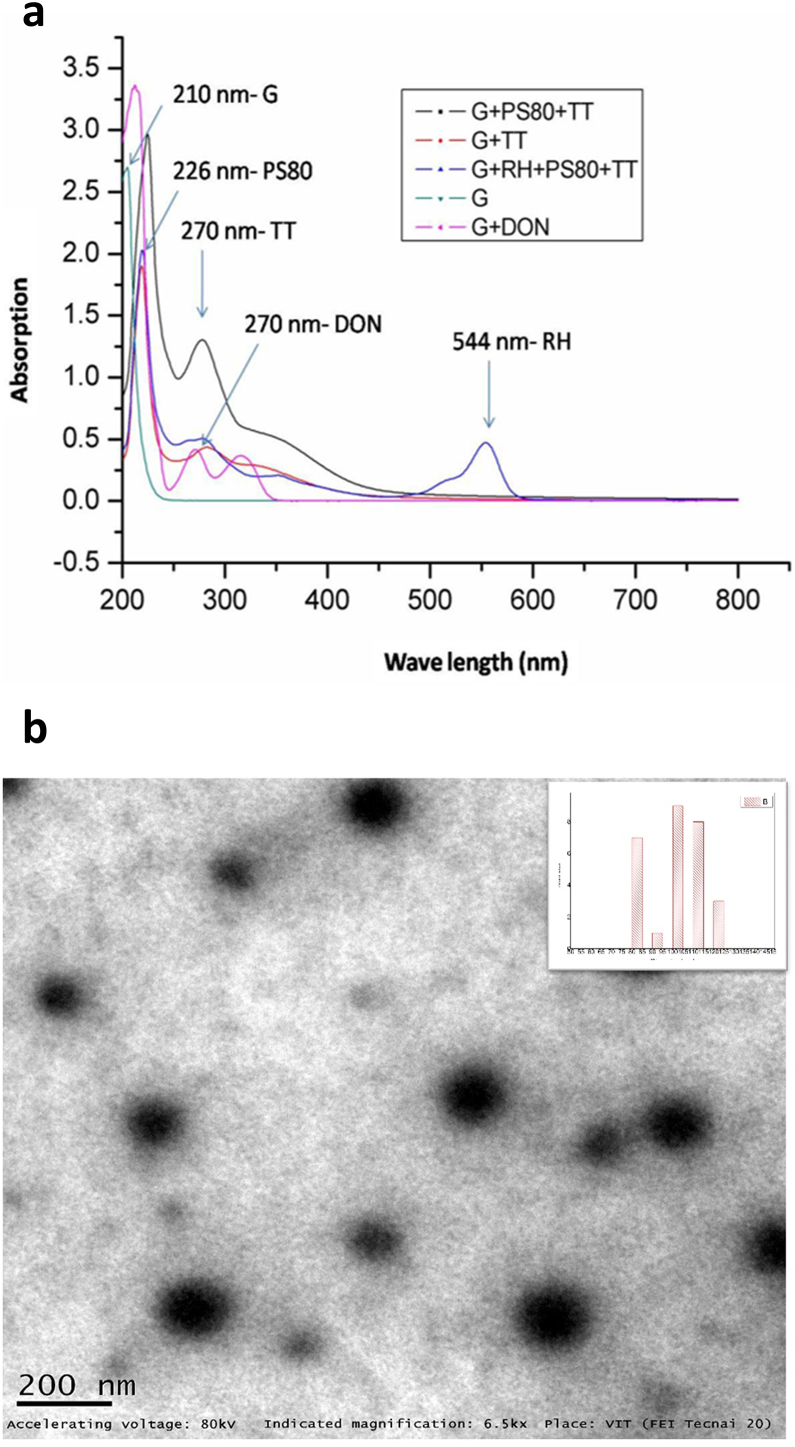
**a.** UV-vis spectrum of the NG with or without conjugated components shows peaks respective of the components. **b.** TEM image of TT loaded NG. The TT loaded NGs diameter increased from 80 nm to 120 nm and were spherical in shape with a smooth surface and good dispersion.

The peaks obtained from the UV spectrum of TT, Rh–B loaded NG, and PS80 coated NG confirmed PS80 and Rh–B were loaded or entrapped inside the NGs. Rh–B was entrapped in PS80 functionalized NG and checked for the ability to cross BBB. The conjugates were purified by size exclusion chromatography (Sephadex). The spectral variations) confirmed the conjugation of NG + TT + PS80 and Rh–B + PS80 + NG. The λmax of NG- 210 nm, RH-B- 544, TT- 269 nm (Figure 2a).

### 3.3 Drug loading-release studies and surface treatment with PS80

The synthesized TT was loaded into NG by post-polymerization. The TT was loaded into the NG and drug loading quality had an encapsulation efficiency of 90%. The difference in NGs size was measured before and after the drug loading by TEM. The TEM results showed that TT loaded NGs diameter increased from 80 nm to 120 nm and were spherical in shape with a smooth surface and good dispersion capability (Figure 2b). The activity *in vitro* drug release was studied under different temperature conditions. TT released from NG was much faster at 37 °C compared to 28 °C or 32 °C (Supplementary Figure 3). This drug release process will play a very important role in delivering targeted drugs via an endocytosis pathway into the brain as mammalian physiological temperature is 37 °C.

### **3.4** Cellular uptake

**Figure 3:**
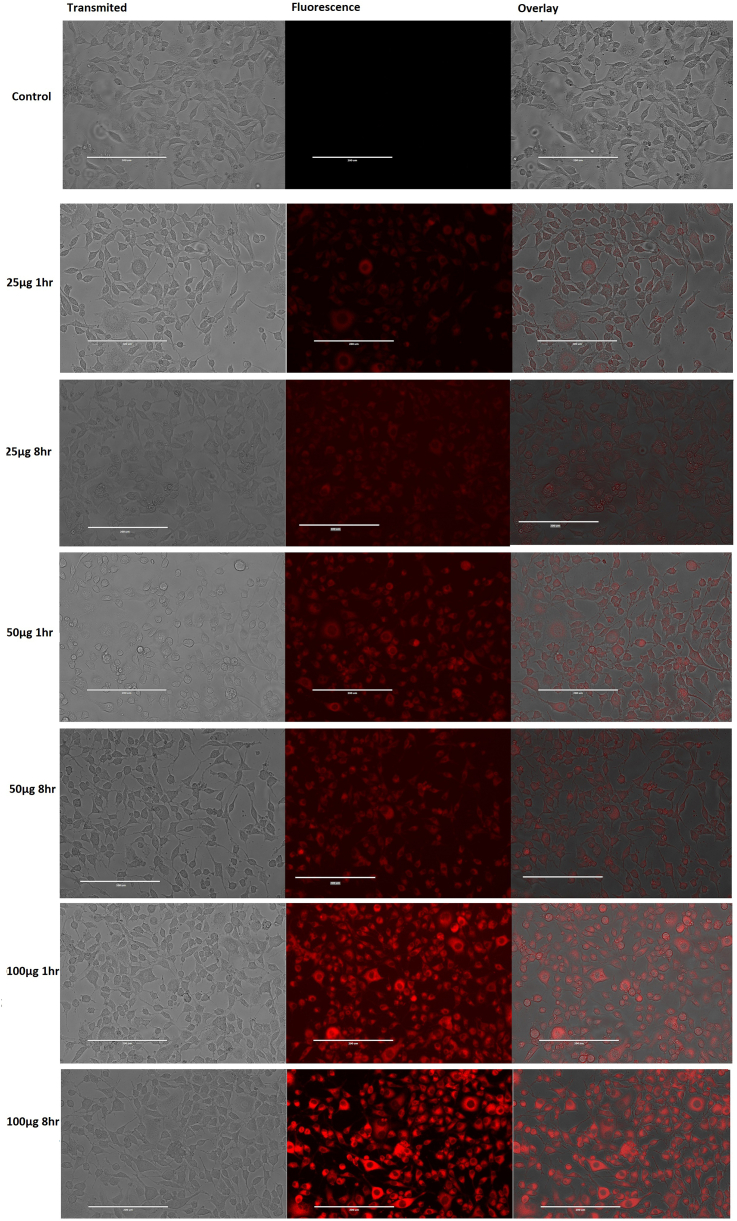
Cellular uptake of NGs in fibroblast cells observed using EVOS inverted microscope.

The NG was covalently tagged with Rh–B to visualize the internalization of NG in fibroblast cells. The most red-fluorescent labeled NGs were distributed in the cytoplasm of fibroblast cells. The intracellular fluorescence of NGs dispersed in aqueous media was compared with Rh–B in DMSO. The cellular uptake EVOS images revealed that NGs have identical *in vitro* cell uptake (Figure 3). These results demonstrated NGs to resolve the barrier of aqueous dispersion by preventing the use of harmful solvents such as DMSO.

### 3.5 Cytotoxicity exhibited by the NG, NG + TT, and TT

The MTT study compared the cytotoxicity of TT in DMSO with NG or NG + TT in fibroblast cell lines. Cytotoxicity was not observed in NG treated fibroblast cells. The NG + TT and TT demonstrated significantly increased cytotoxicity to fibroblast cells after 24 h incubation (Supplementary Figure 4) indicating TT activity remained in the loaded NG.

### 3.6 Zebrafish developmental toxicity

**Figure 4:**
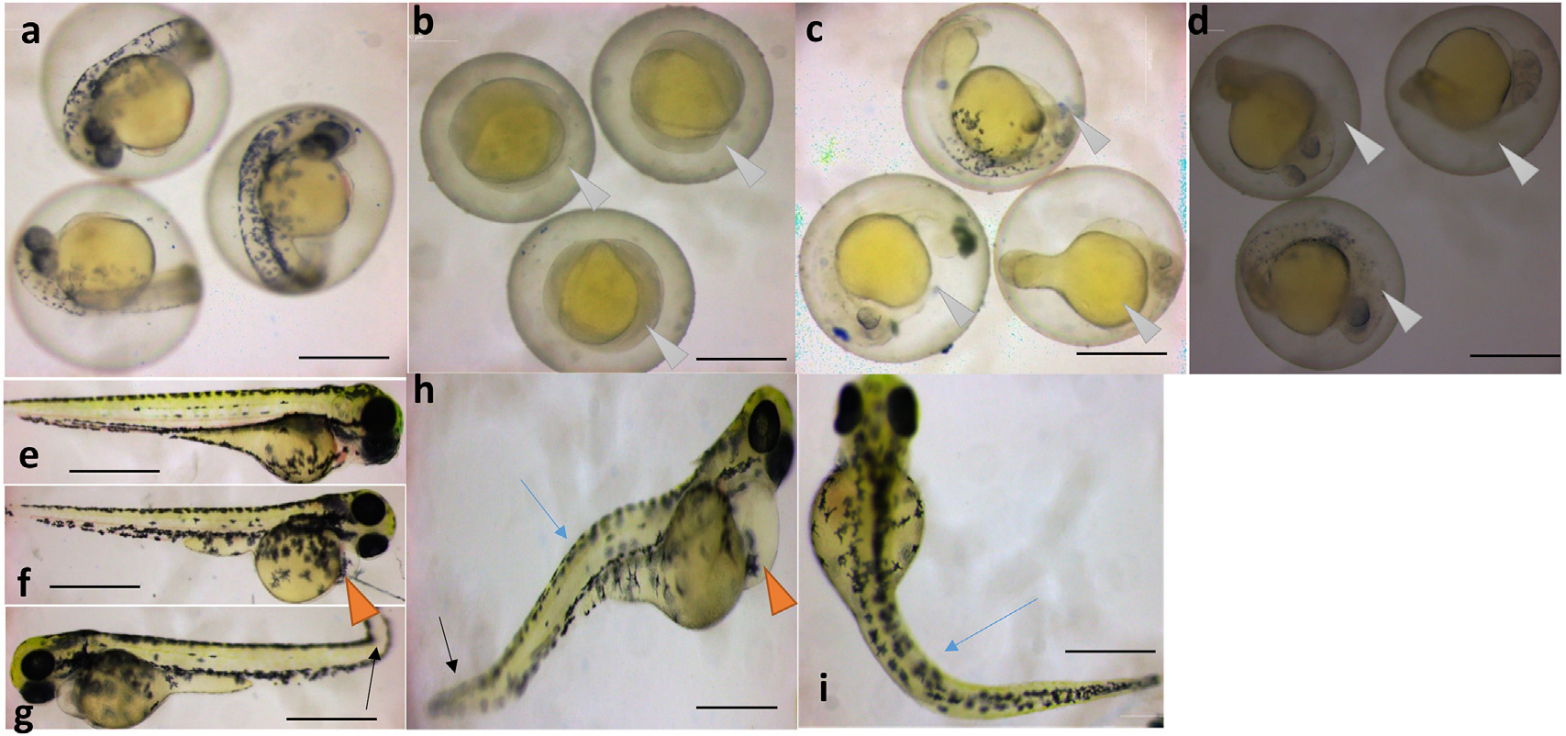
Toxicity evaluation of NG, NG + TT, and TT in developing zebrafish (*Danio rerio*). (a) Control 24 hpf embryo. (b) NG treated embryos at 24 hpf had slow growth at treatments above 300 μg/ml (white arrowhead). NG + TT treated embryos at (c) 24 hpf showed slower growth in the 250 μg/ml treatment group (white arrowhead). TT treated embryos also had a developmental delay at (d) 24 hpf in the 1.2 μg/ml treatment group (white arrow head). (e) 72 hpf control fish. (f) At 72 hpf pericardial edema was observed in the 300 μg/ml NG treatment group (red arrowhead). (g) Bent tail (black arrow) was seen in 72 hpf larvae in the 1.1 μg/ml TT treatment group. (h) At 72 hpf, bent tail (black arrow), spinal kyphosis (blue arrow), and pericardial edema (red arrowhead) were observed in the 200 μg/ml NG + TT treatment group. (i) Spinal kyphosis (blue arrow) was also observed in the 1 μg/ml TT treatment group. Scale bars are 1 mm.

Zebrafish were exposed from 4 - 96 hpf to a range of concentrations of NG, NG + TT, or TT. The acute toxicity (LC_50_) to zebrafish at 96-hpf was 250 μg/ml for NG, 172 μg/ml for NG + TT, and 0.9 μg/ml for TT (Supplementary Figure 5). NG + TT was less toxic in regards to mortality compared to TT. At 24 hpf zebrafish embryos were exposed to 300 μg/ml NG had delayed development and delayed hatching with pericardial edema was observed at 72 hpf (Figure 4). The developmental arrest and early hatching at 24 hpf in the 250 μg/ml treatment group In the NG + TT treatments (Hatching period 72 hpf). Spinal kyphosis, pericardial edema, and bent tail were observed at 72 hpf in the 200 μg/ml treatment group (Figure 4). Slowed growth was observed in the TT treated embryos at 24 hpf in the 1.2 μg/ml treatment group. Similarly spinal kyphosis and bent tail were seen in the 1.1 μg/ml treatment group at 72 hpf **(**Figure 4).

### 3.7 Neurospecificity analysis by microinjection in zebrafish larvae

**Figure 5:**
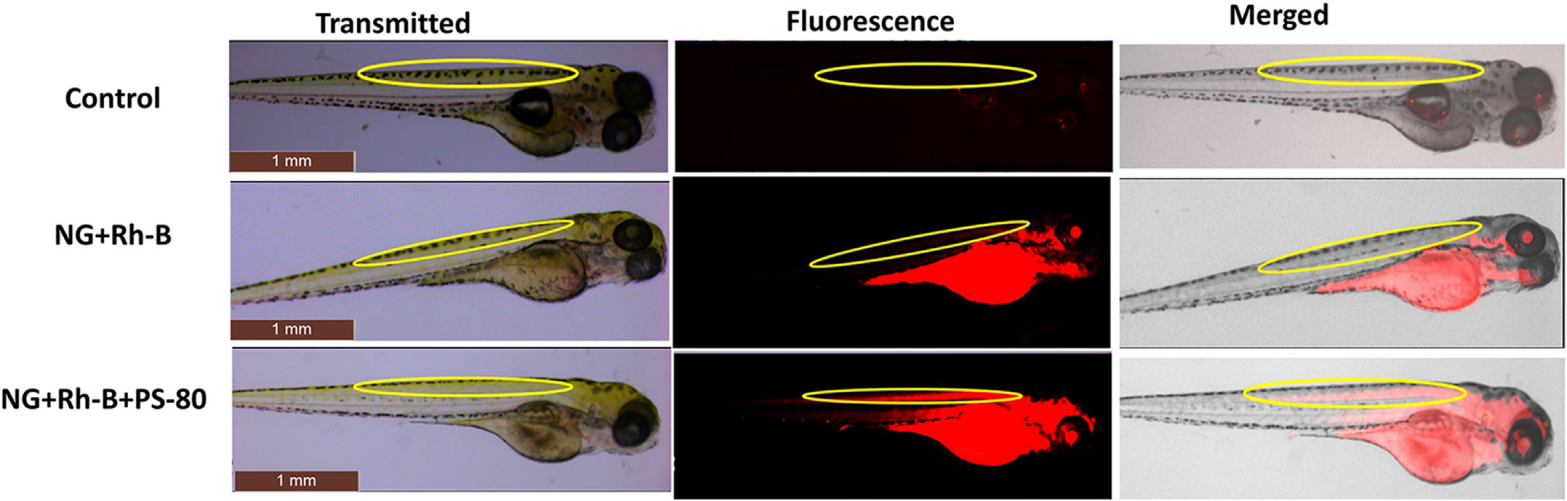
The NG conjugates were microinjected into the aortic region of zebrafish larvae to examine the possibility of crossing the BBB *in vivo* via apolipoprotein receptor-mediated endocytosis. NG + PS80, NG + Rh–B, and NG + Rh–B + PS80 bright field images of microinjected zebrafish larvae (transmitted), fluorescent image (fluorescence), and grayscale + fluorescent merged image (merged). NG + PS80 and NG + Rh–B overlapped image of grayscale and red fluorescence showed no targeted delivery of NG to the CNS due to lack of fluorescence property or inability to cross BBB, respectively. The NG + Rh–B + PS80 overlapped image of grayscale and red fluorescence shows targeted delivery of NG to CNS. The yellow marked region is the spinal cord of zebrafish larvae.

Zebrafish embryos were alive at 24 h after injection (168 hpf) with normal behaviour. NGs were conjugated with Rh–B for fluorescence in larval imaging. After aortic injection of NG + Rh–B bright fluorescence was observed in the body (Figure 5), but no signal was noted in CNS. Our observation proved that NG with 40–60 nm did not enter the CNS. The Rh–B labeled conjugation system (NG + Rh–B + PS80) was injected into the aortic region of zebrafish and fluorescence was seen in the CNS with bright signal in the central canal of CNS (Figure 5). Therefore, the conjugation system of NG + Rh–B + PS80 had crossed the BBB to reach the brain and CNS.

### 3.8 HPLC confirmation of TT delivery in zebrafish brain

**Figure 6:**
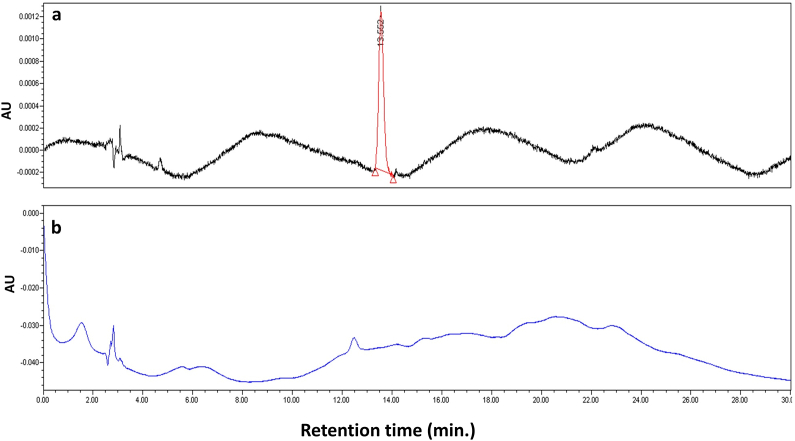
RP-HPLC analysis of TT delivery in adult zebrafish brain. (a) NG + TT with PS80, chromatogram where the TT peak (red) is observed in zebrafish brain at 13.56 min. (b) NG + TT without PS80 treated zebrafish brain, where no TT peak is observed in the chromatogram.

5 μl volumes of NG + TT or NG + TT + PS80 (up to 100 μg/ml) was administered to ensure the entry of 3 μl of TT inside the gastrointestinal tract of zebrafish. Similar to the larval assessments, the NG + TT + PS80 was injected and the delivery of TT was accomplished by the PS80 treated NG overcoming the BBB. Presence of TT in the brain was confirmed by HPLC (Figure 6). The NG + TT + PS80 injected zebrafish brain extract chromatogram observed the TT peak at 13.56 min (Figure 6a), whereas NG + TT injected zebrafish brain extract chromatogram had no TT peak present (Figure 6b).

### 3.9 Behavior analysis

**Figure 7:**
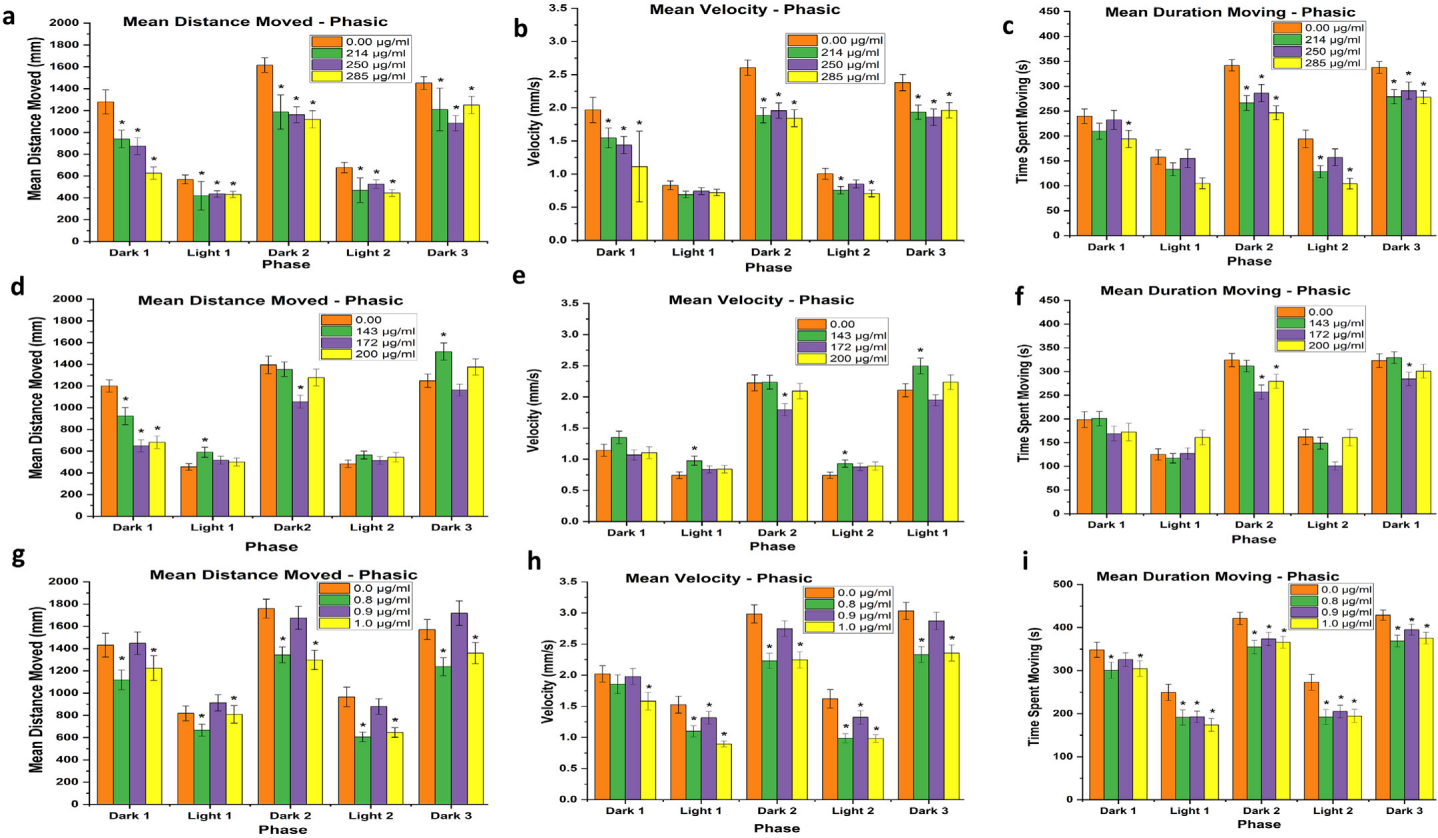
Visual motor response assay in zebrafish larvae at 120 hpf exposed to concentrations below, at, and above the 96 hpf-LC50 for NG (a–c), NG + TT (d–f), or TT (g–i). Error bars represent standard error. ∗p < 0.05, N = 4 with 24 subsamples per treatment in each replicate to total 96 larvae per treatment.

Zebrafish were exposed (4–120 hpf) at concentrations below and above-LC50 concentrations. The NG exposed larvae showed consistent hypoactivity among almost all phases of travelled distance, velocity, and moving time spent. Exceptions included a lack of significant change in light phase 1 for velocity and time spent moving. In addition, no significant differences were observed in time spent moving at the two lower exposure concentrations (214 and 250 μg/ml) in dark phase 1 or at 250 μg/ml in light phase 2 (Figure 7a-c). The NG + TT exposed larvae showed the most minimal and variable behavioral responses among outcomes, phases, and exposure concentrations. Decreased movement was observed for all concentrations in dark phase 1, only at 143 μg/ml in light phase 1, and only at 172 μg/ml in dark phase 2. No significant changes were observed in light phase 2 and an increase in distance travelled or moved was seen in larvae exposed to 143 μg/ml NG + TT (Figure 7d). Alternatively, a different trend was observed for velocity with no significant differences in dark phase 1, but an increase in velocity in both light phases at 143 μg/ml. A decrease in velocity was seen in dark phases 2 and 3, but at 172 μg/ml in dark phase 2 and at 143 μg/ml in dark phase 3 (Figure 7e). Lastly, a significant decrease in time spent moving was observed at 172 and 200 μg/ml in dark phase 2 and only at 172 μg/ml in dark phase 3 (Figure 7f).

The TT exposed larvae showed a more similar trend as observed in the NG exposed larvae in hypoactivity. Specifically, a significant decrease in distance moved was seen in the 0.8 and 1.0 μg/ml treatment groups in all phases (Figure 7g). Alterations in velocity were more variable with a significant decrease in all treatment groups in the two light phases, but only at 1.0 μg/ml in dark phase 1 and at 0.8 and 1.0 μg/ml in dark phases 2 and 3 (Figure 7h). A significant decrease in time spent moving was detected at 0.8 and 1.0 μg/ml in dark phase 1 and in all treatment groups in all other phases (Figure 7i).

### 3.10 Expression analysis of Alzheimer’s disease associated genes

**Figure 8:**
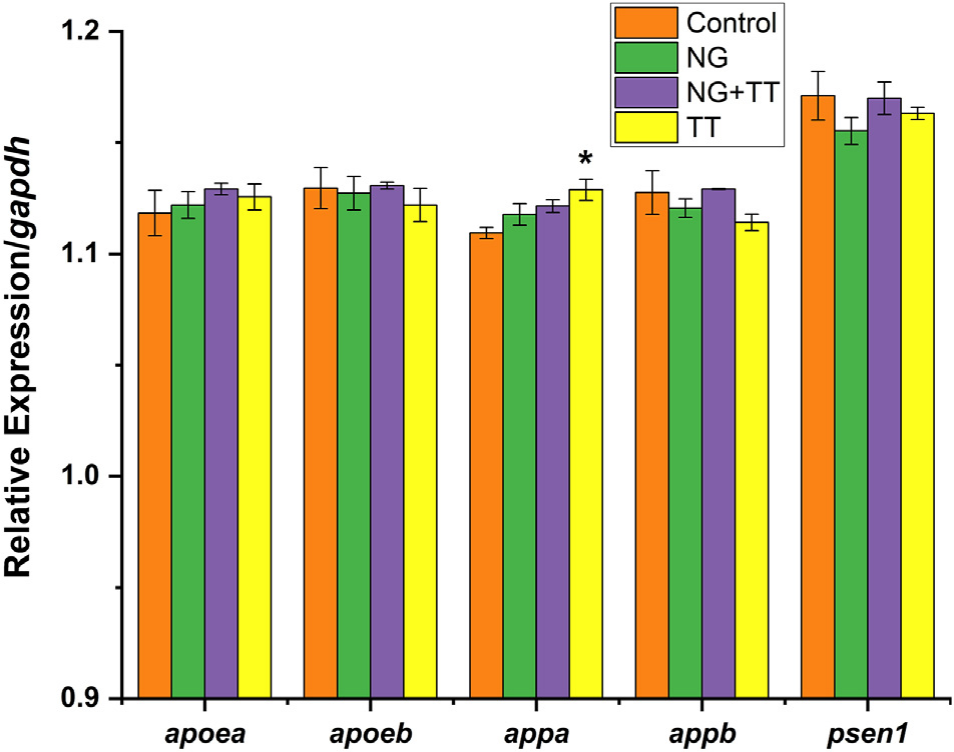
Expression analysis of genes associated with AD (*apoea, apoeb, appab, appb, and psen1*) in the NG, NG + TT, or TT exposed zebrafish larvae at 120 hpf. Error bars indicate standard error from biological replicates in each group. Zebrafish *gapdh* was used as a reference gene. ∗denotes a significant gene expression difference between the control and treatment groups (p < 0.05), N = 3.

The expression of target genes *apoea*, *apoeb*, *appa*, *appb* and *psen1* were determined at 120 hpf following exposure from 4-120 hpf to the 96 hpf-LC50 of NG (250 μg/ml), NG + TT (172 μg/ml), or TT (0.9 μg/ml). Only the expression of *appa* showed a significant increase for the TT treated larvae (p < 0.05). The NG and NG + TT treated larvae had no significant changes in gene expression (Figure 8).

## 4 Discussion

Stimuli responsive drug delivery systems are needed to be designed to distribute neuroactive drugs effectively. Overcoming the BBB and delivering drugs to the brain at therapeutic levels is a big problem in the treatment of neurological disorders [23]. The most popular method of transporting drugs across the BBB is through endogenous transport tools [24]. Recently, PNIPAM-based NGs for controlled drug delivery technologies have been proposed, which can improve drug solubility with prolonged release time and reduce side effects [25]. The synthesized NG has a unique morphology under FE-SEM examination, with a cross-linked and uneven porous structure that is well interconnected to form a mesh-like structure. The network structure of NG is preferable to the microsphere shaped NG for effective drug loading [18]. The temperature-sensitivity of PNIPAM can be used to achieve specific aims and functionalities. In the present study, temperature induced collapse of the PNIPAM NG were aggregated. The amide side chains of PNIPAM blocks form intermolecular hydrogen bonding with the surrounding water molecules [26].

The UV-vis absorption confirmed the conjugation of NG + TT + PS80 and NG + Rh–B + PS80 with an absorption of 270 nm 544 respectively. These findings are similar to past studies reporting a peak of PS80 at 226 nm [27], for Rh–B at 554 nm [28], and for TT at 269 nm [29]. For drug delivery, TT can be quickly loaded into the NG by dissolving TT in the solution at a lower temperature and then raising the temperature to above the LCST for gelation. TT loading was confirmed by TEM and showed an increased size of 80–120 nm, which confirmed the entrapment of TT. The *in vitro* drug release was analyzed and sustained release was observed at 32 °C and 37 °C. Moreover, site-specific drug delivery and controlled drug release can be obtained by maintaining fine control over the temperature. The drug-release kinetics from the NGs was studied with TT. Interactions between these groups and the solvent molecules play dominant roles in determining the temperature-responsive behaviors [30]. Taking the LCST polymers for example, hydrogen bonds can be formed between their hydrophilic groups and water molecules at temperatures below the LCST [31]. The highly hydrated polymer molecules can be well dissolved in water at the molecular level [32]. Drug molecules may be integrated into NGs by chemical conjugation, during polymerization, physical trapping, or physical diffusion methods. Thus the TT was diffused into the NG during its swelling phase [33]. NGs transported the drugs to the CNS by receptor mediated endocytosis [34]. The effective uptake of the NGs by cells were the important feature for therapeutic efficacy [36]. The incorporation of an Rh–B into the NGs provide useful knowledge into uptake and localization. Rh–B was conjugated with NG to check internalization and accumulation in cells for bioimaging in fibroblast cells for cellular uptake due to the presence of caveolae [37]. The caveolae induced endocytosis was the entrance of cells that involve flask-shaped membrane invaginations called caveolae (tiny caves). The results showed that most red-fluorescent labeled NGs were indicating the uptake in the cells NGs. It was hypothesized that uptake may be through the two popular mechanisms of phagocytosis and diffusion via cell walls. Most importantly, after 48 h of incubation, the cells treated with NG + Rh–B did not change in cell morphology, suggesting that they have good biocompatibility.

In this study NG + TT displayed more cytotoxicity toward fibroblast cells than free NG, as assessed by the MTT assay. Previous studies suggested that curcumin nano encapsulation can down-regulate tumor cell proliferation [38]. We noted a change in the number and size of clones between NG + TT and untreated control in concordance with the results of the cytotoxicity study. Thus these findings support the idea of using NG + TT for a continued release of drugs.

The zebrafish was used as a whole animal system to characterize toxicity. A previous study reported that all embryos die at 24 hpf in concentrations greater than 350 μg/ml of the NG and 300 μg/ml of the NG + TT, which are higher than the concentrations we applied in this work. Even though NG, NG + TT, and TT have a narrow range of developmental toxicity in zebrafish embryos, zebrafish exposed to concentrations above the LC50 had an increased incidence of deformities. These results indicate that NG, NG + TT, and TT pose a teratogenic threat to developing zebrafish embryos at these higher concentrations.

The *in vivo* efficacy of NG into the brain was studied in 72 hpf zebrafish larvae with fully developed BBB function similar to human BBB [39] for brain drug delivery studies. NG + Rh–B did not penetrate the BBB in 144 hpf zebrafish larvae, but when NG + Rh–B had the surface treated with PS80, there was significant penetration into the BBB as fluorescence was observed in the CNS. Similarly, the adult fish was orally gavaged with NG + TT or NG + TT + PS80 showed only NG + TT + PS80 was able to deliver TT to the brain. This PS80 is an effective lead substance for brain targeting. In addition, data showed that PS80 coated nanoparticles help to treat neurological disorders [41]. It was noted that zebrafish are poikilothermic and are maintained at a water temperature of 28 °C in standard laboratory conditions. These *in vivo* drug release studies included a short temperature elevation to 37 °C to enhance TT release from the NG.

In the present study, the visual motor response was used at 120 hpf to verify the impact on locomotor activity at 96 hpf-LC50. Behavioral assessments were completed at 120 hpf, because all the organs are differentiated and developed to assess the behavioral movement repertoire [42]. In the visual motor responses test, the NG exposed larvae showed a significant locomotor suppression in all treatment of all phases of the three outcomes assessed (distance moved, velocity, and time spent moving). The NG + TT exposed larvae displayed a biphasic result, dependent on exposure concentration, either stimulating or suppressing locomotive activity. Overall, the NG + TT exposure was the most variable. Similar to the NG treatment groups, the TT treated larvae showed hypoactivity in all phases of each outcome, but there was some variability among TT concentration at which a significant decrease was observed. Interestingly, the most consistent changes were observed in the lowest and highest treatment concentrations. The chemicals can trigger delays in the development of the locomotor and nervous systems, as well as visual impairment in the latter [43]. Hypoactivity can be caused by the presence of malformations [44]. While it was observed that treatment concentrations around the 96 hpf-LC50 could induce malformations, only larvae without malformations were assessed in the visual motor assay to eliminate this confounder.

The molecular changes associated with a neurodegenerative disease, in the present study, quantitative expression of AD associated genes (*appa*, *appb*, *apoea*, *apoeb*, and *psen1*). No significant changes were observed in any of the genes in the larvae exposed to NG or NG + TT. However, the TT treated larvae had an increase in the relative expression of *appa* supporting the novel finding of neuroactive potential of TT. The lack of change in the NG + TT agrees with the lethality assessment in which TT bound to NG limits toxicity. Overall, the zebrafish assessments inform on upper limits of non-toxic thresholds for the NG and for TT. Furthermore, NG + TT toxicity was only completed at 28 °C in the current study to evaluate NG + TT toxicity in standard zebrafish husbandry conditions in comparison to TT. This data can now be used to guide Future studies focused on characterizing NG + TT toxicity profiles in elevated temperature conditions, where release of TT from NG will be enhanced.

## 5 Conclusions

Biocompatible and thermosensitive biodegradable NGs had been developed to achieve targeted drug delivery. The inverse polymerization method was used to synthesize cross-linked PNIPAM polymers. The loading and release of TT was investigated adult zebrafish brain. Intracellular and cytotoxicity studies were carried out in fibroblast cell lines. 144 hpf larval zebrafish a hpf were selected to examine the possibility of transporting NG into the brain. Rh–B + PS80 was covalently conjugated with NG. Further the conjugates were injected into the zebrafish aorta region to examine the distribution of the nanogel and receptor-mediated delivery in the CNS. Real Time larval imaging facilitated the entry of e PS80 with NG into the CNS. Encapsulation of TT within cross-linked NGs showed comprehensive results, justifying the potential use of NG + TT with better solubility, greater cellular uptake, and sustained release. The neurospecific delivery of TT was confirmed in adult zebrafish by oral gavaging of PS80 functionalized NG with TT and analyzed with RP-HPLC. Further toxicity assessment in the zebrafish model addressed behavior and molecular alterations of the NG, NG + TT, and TT. A behavior study in 120 hpf zebrafish larvae revealed that NG and TT exposed larvae showed similar hypoactivity, but that more variability was observed for the TT loaded NG exposed larvae demonstrating both hypo- and hyperactivity. qPCR gene expression analysis showed that NG and NG + TT exposed zebrafish larvae did not influenced the expression of genes associated with AD. Alternatively, zebrafish larvae exposed to TT showed novel significant expression changes in *appa.* Overall, the results suggest PNIPAM NGs have great potential as therapeutic agents for neurological disorders and inform on exposure parameters for future studies.

## Declarations

### Author contribution statement

Pitchai Arjun: Conceived and designed the experiments; Performed the experiments; Analyzed and interpreted the data; Wrote the paper.

Rajaretinam Rajesh Kannan: Conceived and designed the experiments; Contributed reagents, materials, analysis tools or data.

Jennifer L Freeman: Analyzed and interpreted the data; Contributed reagents, materials, analysis tools or data; Wrote the paper.

### Funding statement

Dr. Rajesh Kannan Rajaretinam was supported by Department of Biotechnology, Ministry of Science and Technology [BT/PR6765/NNT/28/618/2012].

Arjun Pitchai was supported by Council of Scientific and Industrial Research, India [09/1205 (0001) 2k18 EMR-I], Science and Engineering Research Board [SB/S9/Z-03/2017-XXV (2018–2019)].

### Data availability statement

Data will be made available on request.

### Declaration of interests statement

The authors declare no conflict of interest.

### Additional information

No additional information is available for this paper.
