# Supplementary material for: Neurospecific fabrication and toxicity assessment of a PNIPAM nanogel encapsulated with trans-tephrostachin for blood-brain-barrier permeability in zebrafish model
Source: Heliyon. 2022 Aug 18;8(8):e10237. doi: 10.1016/j.heliyon.2022.e10237 (PMC9420489; doi:10.1016/j.heliyon.2022.e10237)
Supplement: Supplementary file (1) [file mmc1.docx]

**Supplementary material**

**Neurospecific fabrication and toxicity assessment of a PNIPAM nanogel encapsulated with *trans*-tephrostachin for Blood-Brain-Barrier permeability in zebrafish model**

Pitchai Arjun^1, 2^, [Jennifer L. Freeman](https://www.ncbi.nlm.nih.gov/pubmed/?term=Freeman%20JL%5BAuthor%5D&cauthor=true&cauthor_uid=31151179)^2*^ and Rajaretinam Rajesh Kannan^1*^

^1^Neuroscience Lab, Centre for Molecular and Nanomedical Sciences (CMNS), Centre for Nanoscience and Nanotechnology (CNSNT), School of Bio and Chemical Engineering, Sathyabama Institute of Science and Technology, (Deemed to be University) Jeppiaar Nagar, Rajiv Gandhi Salai, Chennai-600119, Tamil Nadu, India.

^2^ School of Health Sciences, Purdue University, West Lafayette, IN 47907, USA.

*Corresponding authors email: [rajeshnbt12@gmail.com](mailto:rajeshnbt12@gmail.com), [rajeshkannan.icn@gmail.com](mailto:rajeshkannan.icn@gmail.com) (R. Rajesh Kannan), jfreema@purdue.edu ([Jennifer L. Freeman](https://www.ncbi.nlm.nih.gov/pubmed/?term=Freeman%20JL%5BAuthor%5D&cauthor=true&cauthor_uid=31151179)).


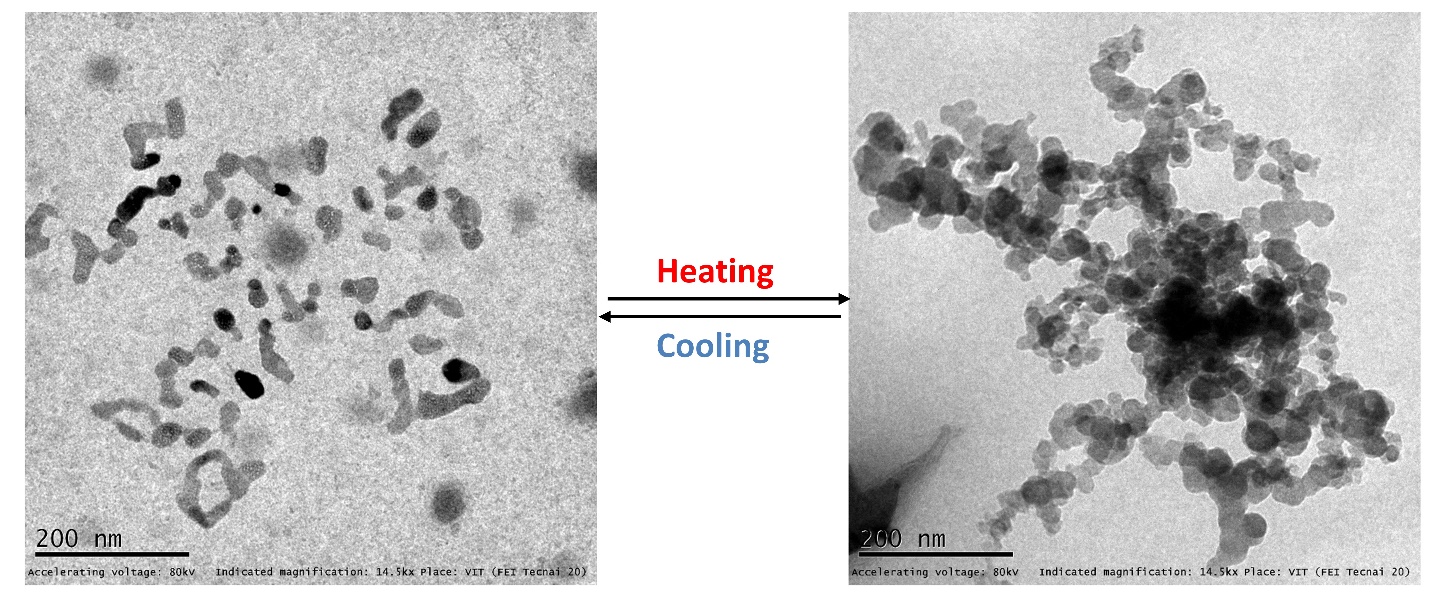


**S. Figure 1** Representative TEM images for the NG at 25 °C (left panel) and at 40 °C (right panel).


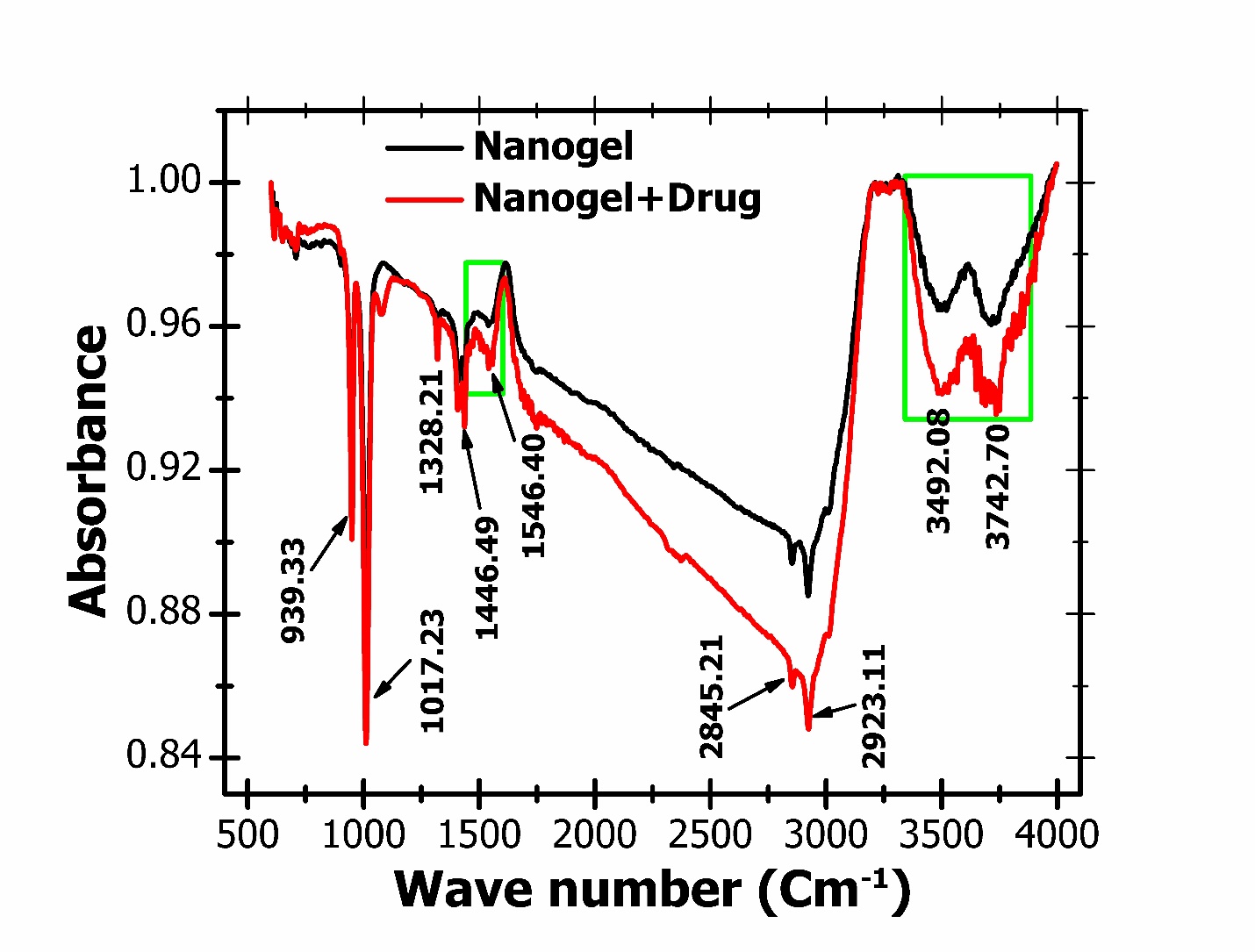


**S. Figure 2** The FTIR spectra for PNIPAM-NG showed expected bands at 3500–3200 cm−1, 1600 and 1500 cm−1, and 2800 and 1400 cm−1 arising from the N–H, C=O, and C–H bonding respectively. These results match well to expected structure of the polymer (black line). The red line clearly indicates the synthesized compound encapsulated in PNIPAM-NGs.


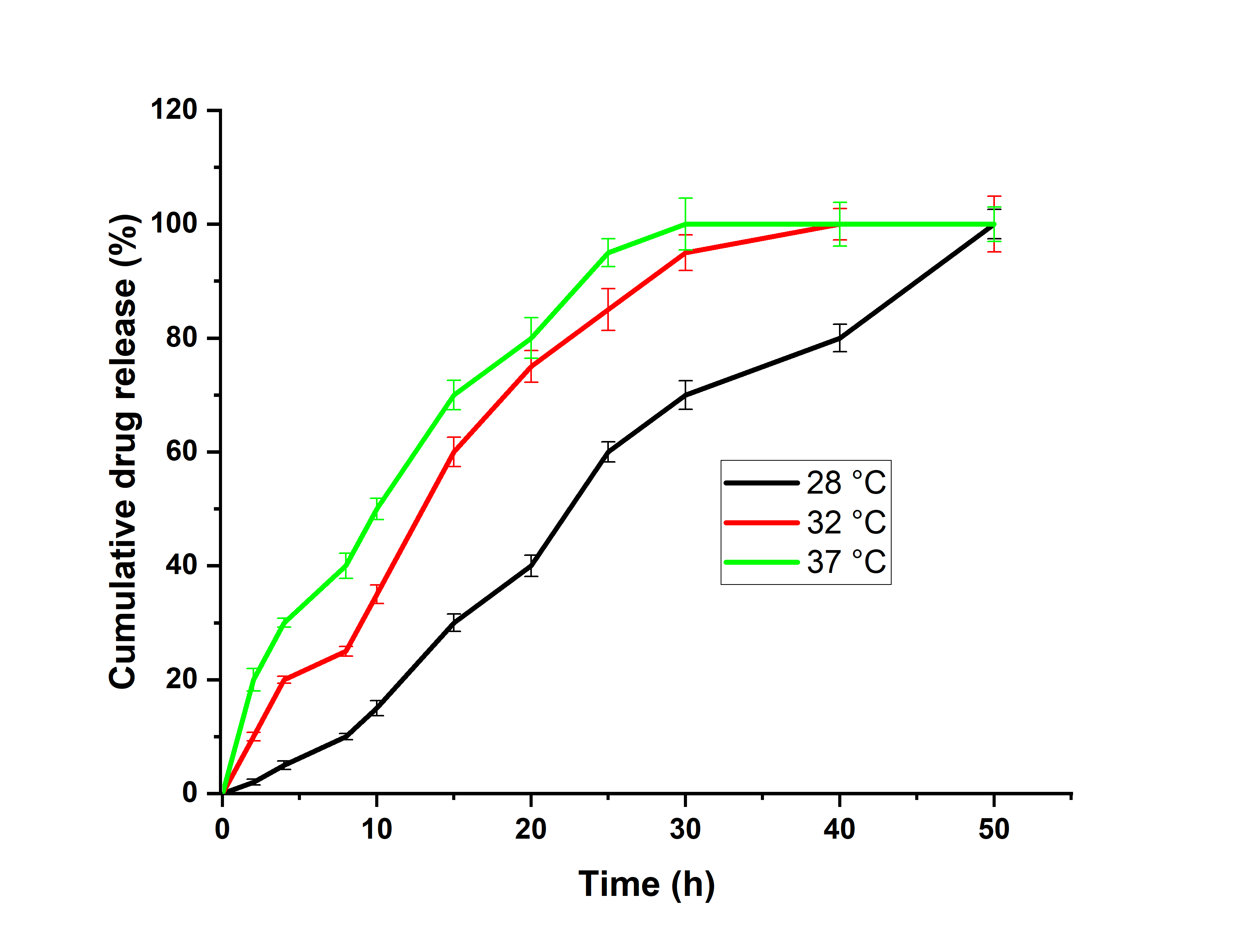


**S. Figure 3** Drug release from thermo-responsive NG at 28°C, 32°C, and 37°C. The better and sustained release was obtained at 32°C and 37°C. Error bars represent SE.


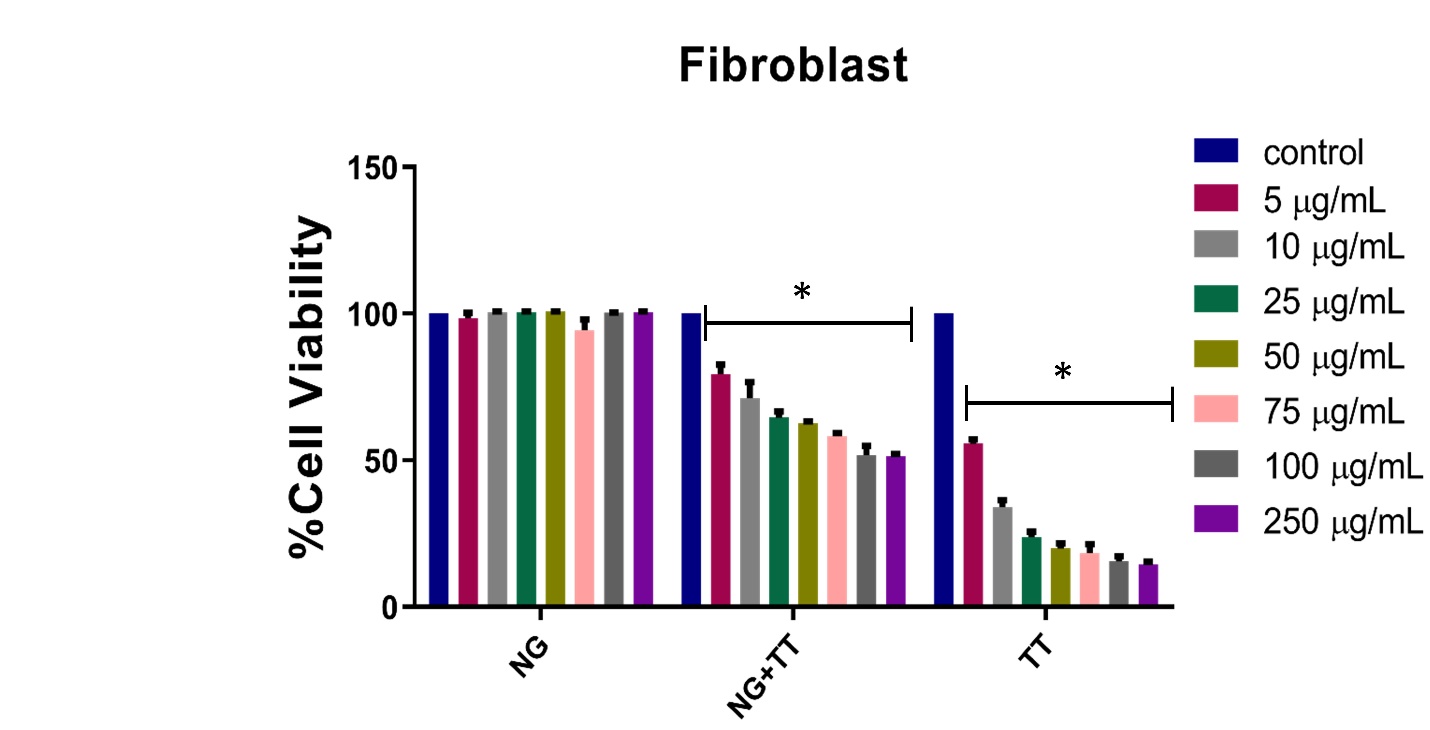


**S. Figure 4** Cytotoxicity of NG, NG+TT, and TT as determined using the MTT assay with fibroblast cells. Error bars represent standard deviation. *p < 0.05, (n = 3).


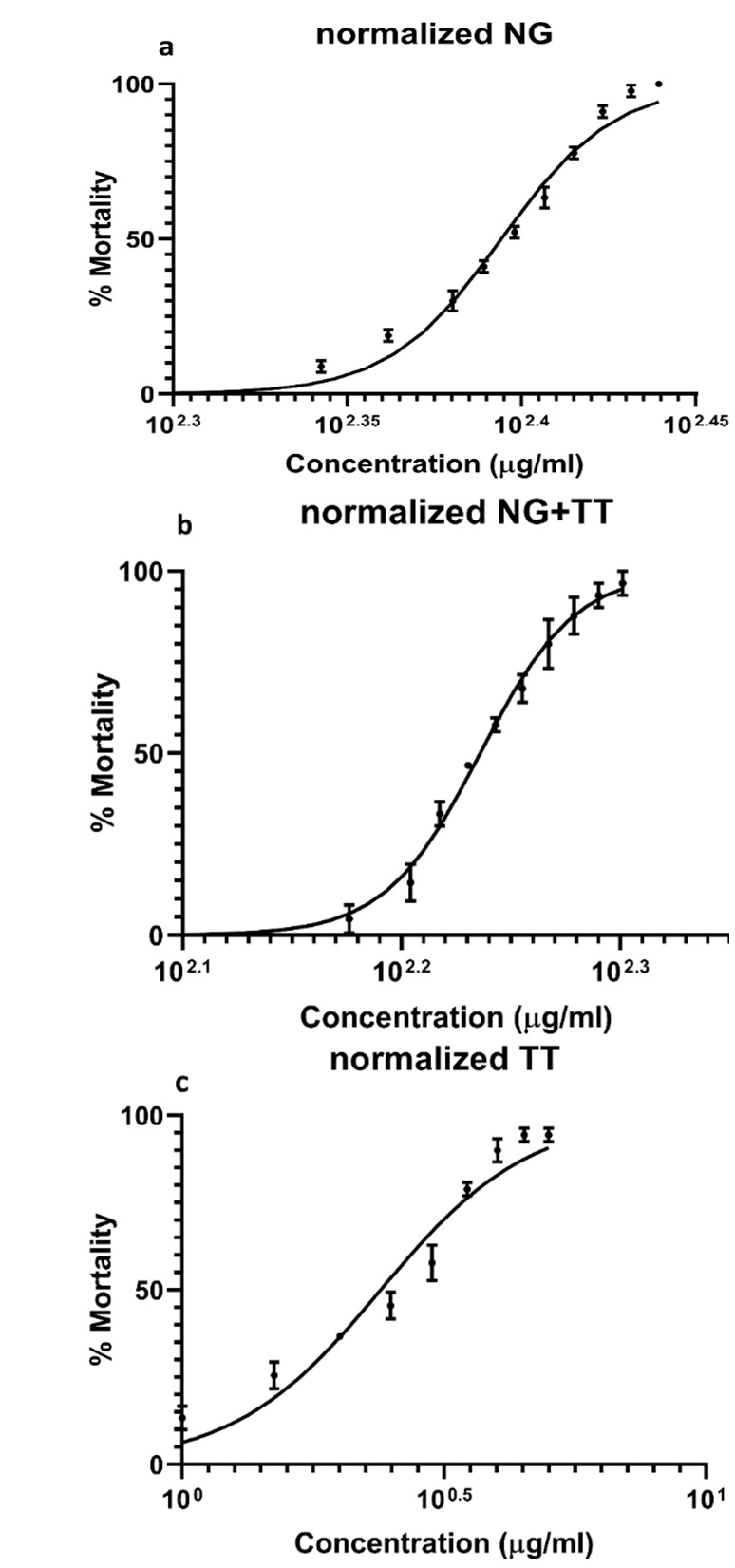


**S. Figure 5** The 96 hpf-LC50 for zebrafish exposed to NG, NG+TT, or TT. The 96 hpf- LC50 was (a) 250 µg/ml for NG, (b) 172 µg/ml for NG+TT, and (c) 0.9 µg/ml for TT. Experiments were performed in triplicate and the data expressed as mean ± SD (n=3).
